# Simultaneous Aortic Valve-in-Valve and Ascending Stent Grafting for Prosthetic Valve Stenosis and Ascending Flap

**DOI:** 10.3400/avd.cr.20-00065

**Published:** 2020-12-25

**Authors:** Yoshiyuki Yamashita, Kazuo Shimamura, Koichi Maeda, Yu Yamada, Toru Ide, Toru Kuratani, Yoshiki Sawa

**Affiliations:** 1 Department of Cardiovascular Surgery, Osaka University Graduate School of Medicine, Suita, Osaka, Japan

**Keywords:** valve-in-valve, ascending stent grafting

## Abstract

Report on total endovascular repair for a diseased aortic valve and the ascending aorta is few. Therefore, we report a case of prosthetic aortic valve stenosis and internal bovine pericardial flap after ascending aortic replacement complicated by congestive heart failure and hemolysis. Because the patient had high surgical risk and was anatomically suitable to undergo ascending endovascular repair, simultaneous transcatheter aortic valve-in-valve implantation and ascending endografting were performed. Her symptoms of heart failure and hemolysis resolved postoperatively. Thus, a simultaneous transcatheter procedure for a diseased aortic valve and the ascending aorta is a feasible option for appropriately selected patients.

## Introduction

Hemolytic anemia is a rare but possible complication both after aortic valve replacement (AVR)^1)^ and ascending aortic replacement.^2,3)^ Reoperative replacement of the aortic valve and the ascending aorta has significant surgical mortality and morbidity, making less invasive approach desirable for high-surgical-risk patients. Here we describe a case of prosthetic aortic valve stenosis and internal bovine pericardial flap in the ascending aorta after surgery for acute aortic dissection complicated by congestive heart failure and hemolytic anemia, which were successfully treated by simultaneous transcatheter aortic valve-in-valve and ascending stent grafting. Written informed consent was obtained from the patient for the publication of this case report and accompanying images.

## Case Report

**Figure 1:**
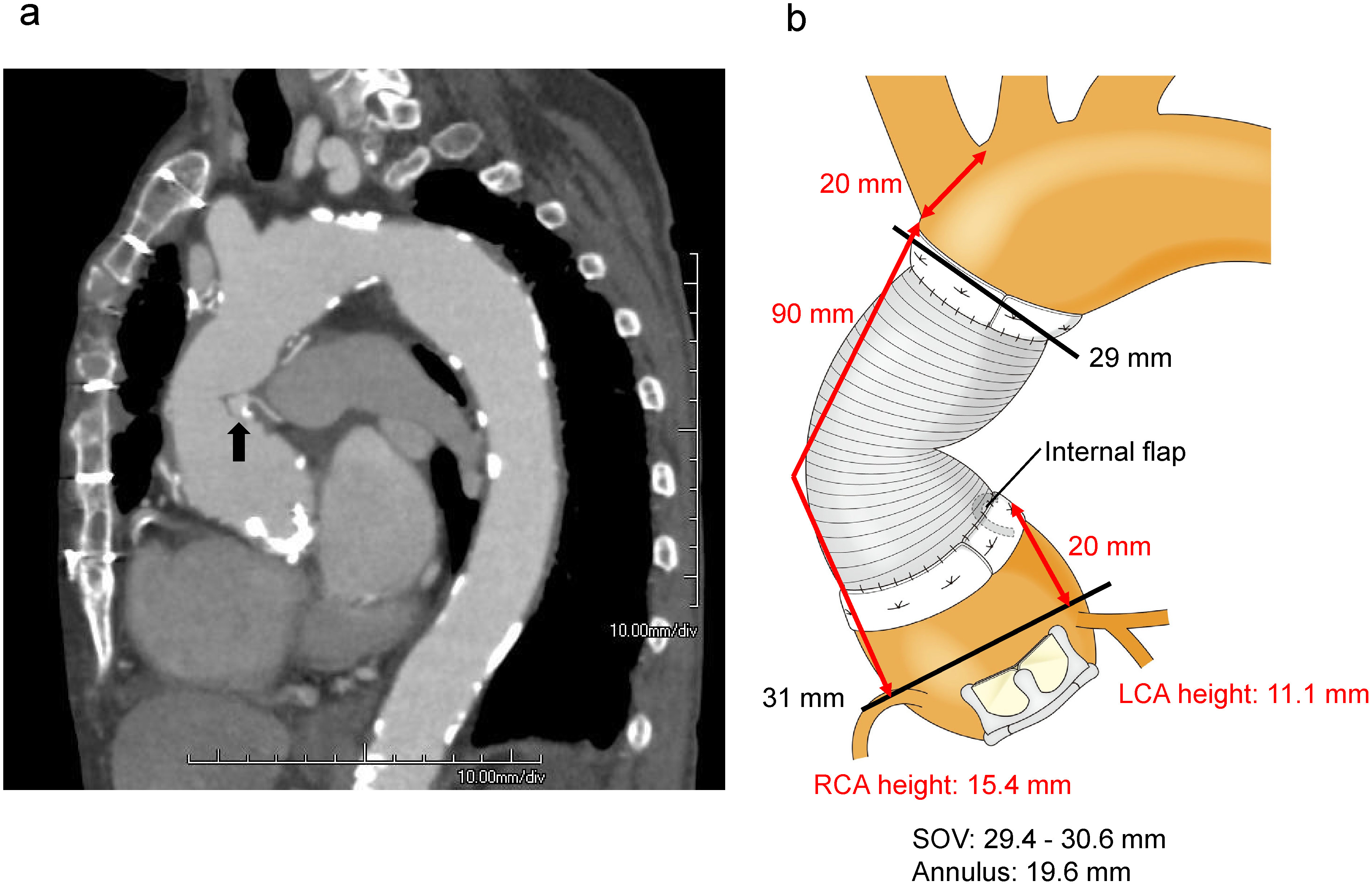
Fig. 1 Computed tomography showing (**a**) a flap (arrow) at the proximal anastomosis site of the ascending aorta, and (**b**) a scheme showing the measurement values of the aortic root complex, ascending aorta, and brachiocephalic artery.

LCA: left coronary artery; RCA: right coronary artery; SOV: sinuses of Valsalva

A 78-year-old woman with bronchial asthma and rheumatic arthritis treated with 5 mg/day oral prednisolone underwent AVR with a 21-mm Carpentier–Edwards Perimount valve (Edwards Lifesciences, Irvine, CA, USA) for aortic stenosis (AS) 12 years earlier and ascending aortic replacement with a 24-mm prosthetic graft for acute aortic dissection 9 years earlier at another institution. During the aortic surgery, the proximal anastomotic sites were reinforced using an inverted bovine pericardium and outer felt strip. Eleven years post-AVR, the patient presented with congestive heart failure and hemolytic anemia, and was referred to our institution. The laboratory values showed hemoglobin of 7.6 g/dl, lactate dehydrogenase (LDH) of 1221 IU/l, low haptoglobin, and the presence of fragmented red cells in the blood smear. Transthoracic echocardiography showed severe prosthetic valve stenosis with a peak velocity of 4.1 m/s and mean pressure gradient of 41 mmHg. Additionally, computed tomography (CT) revealed a flap at the proximal anastomotic site of the ascending aorta (**Fig. 1a**), which was a suspected disrupted bovine pericardium. Although transesophageal echocardiography and four-dimensional CT failed to demonstrate the accelerated blood flow in the flap, based on the previous reports,^2,3)^ this flap was considered one possible cause of hemolysis, as well as the prosthetic valve stenosis. Initially, redo open AVR and ascending aortic repair were planned. However, given her severe comorbidities and the estimated high surgical risk (Society of Thoracic Surgery Predicted Risk of Mortality Score: 11.5%), we evaluated the possibility of total endovascular repair. Exact measurements using electrocardiogram-gated multidetector-row CT showed that the sinotubular junction to the flap was approximately 20 mm, which was considered to be acceptable in length, as the proximal sealing zone, to exclude the flap (**Fig. 1b**). Given the other measurement values, including the outer curvature length from the sinotubular junction (proximal landing zone) to the brachiocephalic artery (BCA) of 90 mm, diameter of the sinotubular junction of 31 mm, and diameter of the distal ascending aorta of 29 mm, we deemed that ascending stent grafting using the shortest Conformable GORE® TAG® endoprosthesis with ACTIVE CONTROL system 37–100 (WL Gore & Associates, Flagstaff, AZ, USA) would be feasible. Regarding the valve-in-valve procedure, a 23-mm EvolutR valve (Medtronic, Minneapolis, MN, USA) was selected according to the internal diameter of the prosthetic aortic valve, which is 19.6 mm. Based on the measurement values, including the diameter of sinuses of Valsalva of 29.4–30.6 mm, and the right and left coronary height from the annulus of 15.4 and 11.1 mm, respectively (**Fig. 1b**), a risk of coronary obstruction after valve-in-valve procedure was considered to be minimal. Therefore, our heart and aortic team decided to perform simultaneous transcatheter repair for symptomatic severe prosthetic aortic valve stenosis and ascending aortic flap.

**Figure 2:**
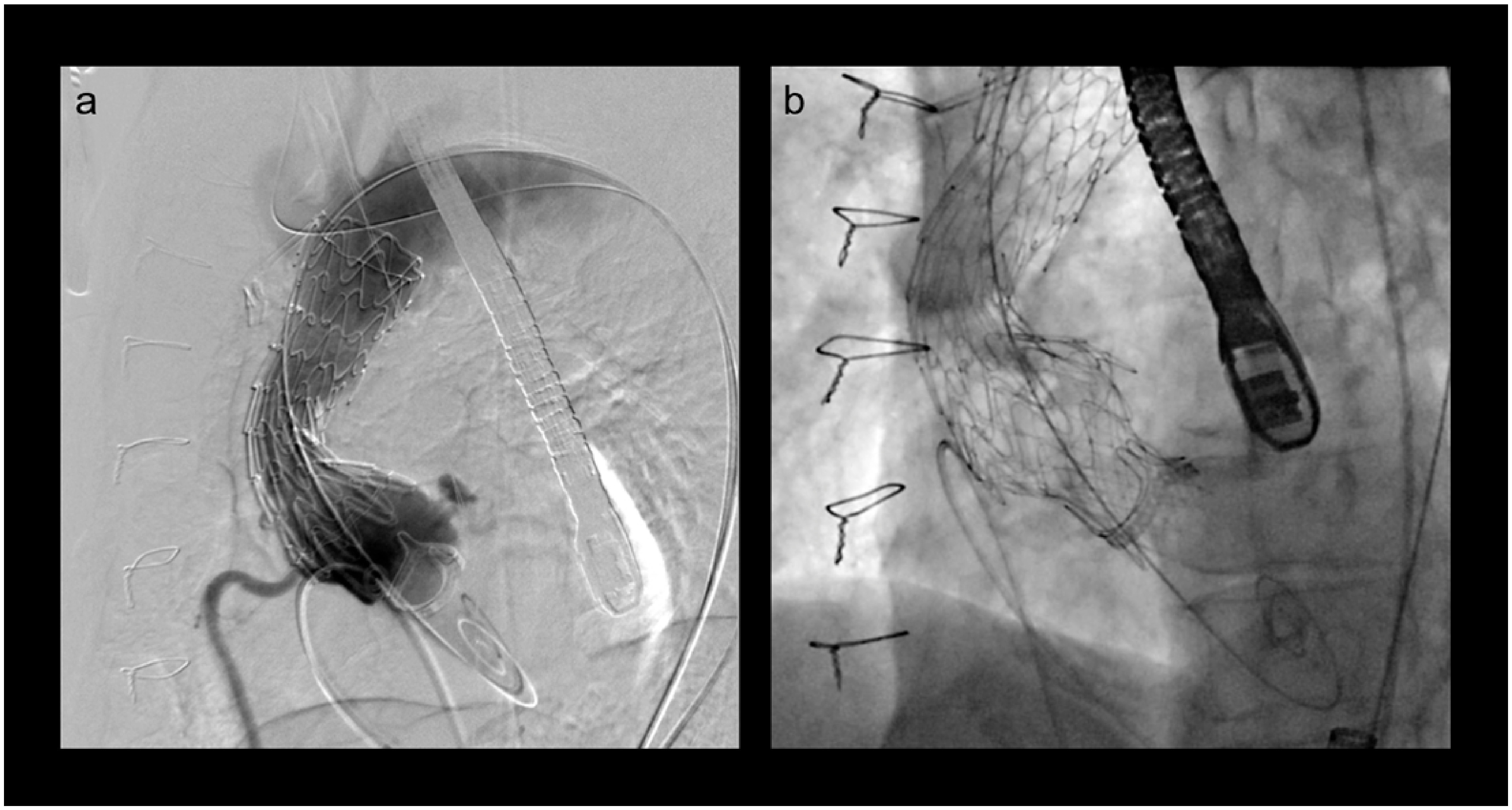
Fig. 2 Fluoroscopy showing (**a**) the implanted stent graft in the ascending aorta and (**b**) the EvolutR (Medtronic, Minneapolis, MN, USA) in valve-in-valve fashion.

**Figure 3:**
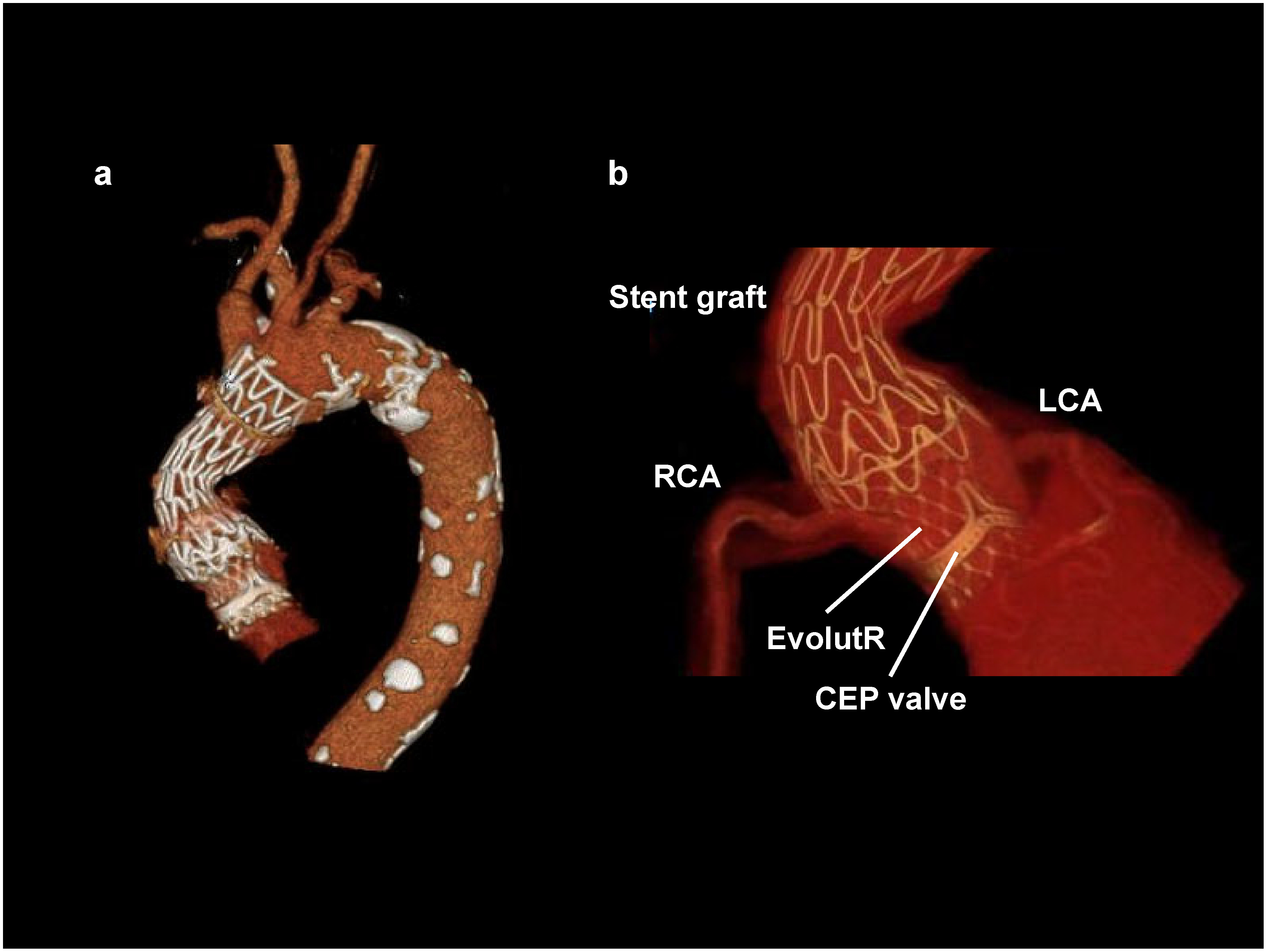
Fig. 3 Postoperative computed tomography showing the appropriately positioned aortic prosthetic valve and the stent graft in the ascending aorta with the patent (**a**) supra-aortic arch vessels and (**b**) coronary arteries.

CEP: Carpentier–Edwards Perimount; LCA: left coronary artery; RCA: right coronary artery

Under general anesthesia, a preemptive guidewire protection was undergone via the right carotid artery in advance so that eventual stent implantation in the BCA could be performed. Next, a Safari guidewire (Boston Scientific, Minnetonka, MN, USA) was passed through the aortic valve via the femoral artery. After angiographically confirming the ostium of the coronary arteries and BCA, we advanced the stent graft into the ascending aorta (**Fig. 2a**) and deployed it in a two-stage manner.^4)^ In detail, the device was placed in the greater curvature of the ascending aorta by pushing the guide wire, and half deployment was performed in a position where it was 10 mm proximal to the intended final proximal landing zone. Then, after confirming the right coronary artery ostium by angiography, position adjustment was made by pulling back the device, and full deployment was finalized. As expected, the orifice of the BCA was partially covered by the distal end of the stent graft; however, there was no pressure gradient between the upper extremities. Subsequently, a 23-mm EvolutR valve was successfully deployed in valve-in-valve fashion (**Fig. 2b**). Postoperative CT confirmed the appropriately positioned EvolutR valve and stent graft with good exclusion of the flap (**Fig. 3**).

The patient was extubated in the operation room. She had no interarm blood pressure differences nor signs of ischemia of the right arm or brain, and her postoperative course was uneventful. CT confirmed the appropriately positioned EvolutR valve and stent graft with good exclusion of the flap (**Fig. 3**). Echocardiography revealed a mean pressure gradient across the aortic valve of 10 mmHg with trivial perivalvular leakage. Her LDH decreased to 346 IU/l, and 10 months postoperatively, hemolytic anemia has not recurred.

## Discussion

A simultaneous valve-in-valve and ascending stent grafting were performed in a patient with a high open surgical risk for several reasons. First, we could not determine whether the AS or the ascending aortic flap was the exact cause of the hemolysis. Hemolysis after aortic surgery has been reported in several studies.^2,3)^ Inverted internal felt strips and severely kinked grafts, which cause accelerated flow and mechanical damage to red blood cell, are the main causes of hemolysis.^2,3)^ In contrast, significant AS notably reduced hemoglobin and haptoglobin levels and increased LDH levels,^5)^ indicating that AS also potentially causes hemolysis.^1,5)^ Second, the patient had symptomatic severe AS requiring re-AVR, of which staged stent grafting following implantation of the EvolutR valve, which was the only approved valve for valve-in-valve in Japan at that time, could not be performed because the high frame of the EvolutR valve would interfere with the proximal landing zone. Therefore, we considered it reasonable to perform simultaneous intervention for prosthetic valve deterioration and ascending flap to treat both the congestive heart failure and possible causes of the hemolysis.

To date, ascending stent grafting has been performed only in patients with high surgical risks.^6,7)^ This is partly because anatomical characteristics make ascending stent grafting challenging, especially for lesions in the proximal ascending aorta, because proximity to the aortic valve and coronary ostia can lead to aortic insufficiency or coronary occlusion.^6,7)^ In addition, distally, stent migration can lead to BCA occlusion. To prevent these critical complications, precise planning and accuracy in deploying stent graft are crucial for adequate exclusion of the treated disease. Although our patient had a flap in the proximal aorta, optimal outcomes were achieved by appropriate planning based on exact measurements of the involved structures using electrocardiogram-gated multidetector-row CT and precise deployment of the Conformable GORE® TAG® endoprosthesis with ACTIVE CONTROL system. This system offers an optional angulation mechanism of the proximal stent end and two-stage deployment for more controlled device positioning.^4,8)^ Optional angulation control can be beneficial in an angulated part of the aorta and in reducing the risk of proximal bird-beak effect and endoleak. The two-stage deployment allows the stent graft to be opened to its intermediate diameter, allowing repositioning of the stent graft to facilitate better positioning and correction. Of note, this feature permits continuous blood flow through the stent graft lumen and around the device, which is especially important in more proximal landing zones, where blood pressure dynamics are more demanding, and does not require aggressive blood pressure reduction such as rapid ventricular pacing. Using Conformable GORE® TAG® endoprosthesis with ACTIVE CONTROL system can facilitate in the accurate deployment and conformability of the stent graft in the ascending aorta, minimizing the risk of inadvertent coverage of coronary arteries or supra-aortic branches. However, precise deployment requires adequate understanding of the stent graft behavior during the deployment. In addition, the available shortest size for this stent graft is 10 cm in length, and its application to the ascending aorta is still limited. We expect that developments in ascending stent grafts will advance treatment options for ascending aortic pathologies.

## Conclusion

A simultaneous transcatheter procedure for a diseased aortic valve and ascending aorta is a feasible option for appropriately selected patients.
